# p53-Independent Effects of Set7/9 Lysine Methyltransferase on Metabolism of Non-Small Cell Lung Cancer Cells

**DOI:** 10.3389/fonc.2021.706668

**Published:** 2021-10-06

**Authors:** Alexandra Daks, Oleg Shuvalov, Olga Fedorova, Alexey Petukhov, Larissa Lezina, Arsenia Zharova, Ekaterina Baidyuk, Alexander Khudiakov, Nickolai A. Barlev

**Affiliations:** ^1^ Institute of Cytology, Russian Academy of Sciences, St Petersburg, Russia; ^2^ Institute of Molecular Biology and Genetics, Almazov National Medical Research Centre, St Petersburg, Russia; ^3^ Regulation of Cell Signaling Laboratory, Moscow Institute of Physics and Technology, Dolgoprudny, Russia

**Keywords:** Set7/9, SETD7, non-small cell lung cancer (NSCLC), glycolysis, metabolism

## Abstract

Set7/9 is a lysine-specific methyltransferase, which regulates the functioning of both the histone and non-histone substrates, thereby significantly affecting the global gene expression landscape. Using microarray expression profiling, we have identified several key master regulators of metabolic networks, including c-Myc, that were affected by Set7/9 status. Consistent with this observation, c-Myc transcriptional targets—genes encoding the glycolytic enzymes hexokinase (HK2), aldolase (ALDOB), and lactate dehydrogenase (LDHA)—were upregulated upon Set7/9 knockdown (Set7/9KD). Importantly, we showed the short hairpin RNA (shRNA)-mediated attenuation of Set7/9 augmented c-Myc, GLUT1, HK2, ALDOA, and LDHA expression in non-small cell lung cancer (NSCLC) cell lines, not only at the transcriptional but also at the protein level. In line with this observation, Set7/9KD significantly augmented the membrane mitochondrial potential (MMP), glycolysis, respiration, and the proliferation rate of NSCLC cells. Importantly, all these effects of Set7/9 on cell metabolism were p53-independent. Bioinformatic analysis has shown a synergistic impact of Set7/9 together with either GLUT1, HIF1A, HK2, or LDHA on the survival of lung cancer patients. Based on these evidence, we hypothesize that Set7/9 can be an important regulator of energy metabolism in NSCLC.

## Introduction

Lysine methylation plays an important role in global transcription regulation. Depending on the location of the target lysine in histone tails, this post-translational modification can either promote or repress transcription by affecting the architecture of chromatin. In addition, lysine methylation, by competing with other lysine-specific modifications (e.g., acetylation, ubiquitinylation, and SUMOylation), can affect the protein stability and hence its functioning (1). Set7/9 (alternative name SETD7) is a SET [Su(var)-3–9, Enhancer-of-Zeste, Trithorax] domain-containing protein that utilizes both histone and non-histone proteins as substrates. Initially, Set7/9 was shown to specifically monomethylate lysine 4 of histone 3 (H3K4me1), which is a positive mark for transcriptional activation (2, 3).

For instance, Set7/9-mediated methylation of histone H3 at lysine 4 enhances the transcriptional activation of myogenic differentiation genes (MYOD, MYOGENIN, MHC, and MCK) (4), the inflammatory gene RelA/NFκB (5), nitric oxide synthase (NOS2) (6), SREK1IP1, and PGC (7). Later, in addition to histone H3, Set7/9 was shown to methylate histones H2A, H2B, and H1.4 (8–10).

Several examples of non-histone substrates of Set7/9 include the tumor suppressor p53 (11), estrogen receptor alpha (ERa) (12), PCAF (P300/CBP-associated factor) (13), RelA (NFκB) (14), β-catenin (15), and E2F1 (16). By methylating these substrates, Set7/9 regulates their activity, stability, and subcellular localization (8).

Set7/9 is shown to be involved in various signaling pathways associated with several diseases, including cancer (8). We and other researchers have demonstrated the involvement of this lysine methyltransferase in the progression of various malignancies, including lung cancer (16–19). Lung cancer is one of the most frequent and dangerous groups of malignancies. It tops the list of cancer-related deaths in men and ranks second in women worldwide (20). The 5-year survival rate with all types of lung cancer ranges between only 17% and 21%. Importantly, the role of Set7/9 in lung cancer is rather contradictive, since it was shown to have both oncogenic (16, 18) and tumor-suppressive properties (21).

Using RNA-seq analysis, Keating with co-authors (22) have revealed a correlation between the gene expression profile in Set7/9 knocked down cells and the status of several transcription factors known to be the targets of Set7/9, including p53, NFκB, c-Jun, c-Fos, GATA2, ERa, and STAT3. This study linked the cellular status of Set7/9 with global changes in gene expression profiles of various cell lines.

To further elucidate the role of Set7/9 in tumorigenesis, we assessed the effect of Set7/9 ablation on global gene expression in various cancer cell lines. We have identified c-Myc and HIF1A as Set7/9-dependent genes. Moreover, we have shown that NSCLC cells with an attenuated expression of Set7/9 displayed increased mitochondrial membrane potential (MMP), glycolysis, and respiration rates. In line with these observations, Set7/9-deficient cells possessed elevated proliferation rates. Bioinformatic analysis revealed the synergistic effect between the Set7/9 and either HIF1A, HK2, GLUT1, or LDHA expression levels on the survival outcome of lung cancer patients.

## Materials and Methods

### Cell Cultures and Stable Cell Lines Establishment

All cell lines were purchased from American Type Culture Collection (ATCC) (USA) and genotyped by the shared research facility “Vertebrate cell culture collection,” Institute of Cytology, Russian Academy of Science, St Petersburg, Russia. Cells were incubated in either Dulbecco’s modified Eagle’s medium (DMEM) (U2OS osteosarcoma cells) or Roswell Park Memorial Institute (RPMI) (H1299, A549, and H1975) media, supplemented with 10% fetal bovine serum (FBS) (Gibco, USA), and 50 µg/ml gentamicin.

U2OS cells with tetracycline-inducible (Tet-on) expression of short hairpin RNA (shRNA) against Set7/9 (pSuperior-shRNA-Set7/9 or U2OS Set7/9 KD) and the reference cell line (U2OS pSuperior) were generated as described previously (16).

To establish lung cancer cell lines (H1299 and H1975) with stable Set7/9 knockdown, the lentiviral transduction by pLKO1.puro shRNA Set7/9 or scramble was carried out as described (23). The following specific shRNA oligonucleotides were annealed prior to cloning into the pLKO.1-TRC vector digested with AgeI and EcoR1 enzymes: top, 5′-CCGGGATCTATGCACTACGTTTATCCTCGAGGATAAACGTAGTGCATAGATCTTTTT-3′ and bottom, 5′-AATTAAAAAGATCTATGCACTACGTTTATCCTCGAGGATAAACGTAGTGCATAGATC-3′.

H1299 cell lines with tetracycline-inducible (Tet-on) expression of wild-type p53 (p53wt) and mutant p53 R273H mutation (p53mut) and a control cell line (ctrl) (a kind gift of Dr P. Muller, University of Leicester, UK) were used to knockdown Set7/9.

Set7/9 knockout in A549 cells was generated using CRISPR/Cas9 technology. Guide RNA (5′-TAGCGACGACGAGATGGTGGAGG-3′) specific to Set7/9 was cloned into lenti_V2.0 vector (Addgene) according to the manufacturer’s instructions. A549 cells were transfected by Lipofectamin 2000 (Invitrogen, USA) with either a vehicle (lenti_V2.0 vector) or a vector encoding for Set7/9-specific gRNA, followed by 3 days selection with puromycin (5 µM).

### Western Blotting

For Western blot analysis, whole-cell extracts were prepared using radioimmunoprecipitation assay (RIPA) buffer. The primary antibodies used against the analyzed proteins were as follows: Set7/9 (1:1,000, 2813, Cell Signaling), β-actin (1:5,000, A3854, Sigma-Aldrich, USA), HK2 (1:1,000, B-8, Santa Cruz Biotechnologies, USA), c-Myc (1:500, 9402S, Cell Signaling, USA), HIF1A (1:1,000, ab16066, Abcam, USA), ALDOA (1:1,000, sc-377058, Santa Cruz Biotechnologies, USA), and LDHA 1:1,000, 2012S, Cell Signaling, USA). The secondary antibodies used were antimouse and anti-rabbit (1:10,000; Sigma-Aldrich, USA). Normalization of Western blot signals was performed based on the ratio between pixel intensities of the bands corresponding to target proteins and loading controls (β-actin), respectively. Densitometry was carried out using the application of Bio-Rad Image Lab software.

### Real-Time PCR

Total RNA was extracted from cells using TRIzol Reagent (Invitrogen, USA) according to the manufacturer’s instructions. Two micrograms of total RNA were reverse transcribed to complementary DNA (cDNA) with oligo d(T) primer using a RevertAid First-Strand cDNA Synthesis Kit (Evrogen, Russia). cDNAs were amplified by real-time PCR on a CFX 1000 PCR machine (BioRad, USA) using SYBR green mix (Evrogen, Russia) in triplicate. Data were analyzed by CFX Manager software. Relative amounts of SLC19A1, Hk2, ALDOA, LDHA, c-Myc, and HIF1A messenger RNAs (mRNAs) were normalized to β-actin mRNA. The following primers were used: SLC2A (GLUT1), forward 5′-AAGGTGATCGAGGAGTTCTACA-3′ and reverse 5′-ATGCCCCCAACAGAAAAGATG-3′; HK2, forward 5′-AAGGCTTCAAGGCATCTG-3′ and reverse 5′-GCCAGGTCCTTCACTGTCTC-3′; ALDOA, forward 5′-CGGGAAGAAGGAGAACCTG-3′ and reverse 5′-CCACAGGTCATCATAGTTCC-3′; LDHA, forward 5′- AGCCCGATTCCGTTACCT-3′ and reverse 5′-AGCCCGATTCCGTTACCT-3′; HIF1A, forward 5′-CATAAAGTCTGCAACATGGAAGGT-3′ and reverse 5′-ATTTGATGGGTGAGGAATGGGTT-3′; Myc (c-Myc), forward 5′-CTCCTCCTCGTCGCAGTAGA-3′ and reverse 5′-GCTGCTTAGACGCTGGATTT-3′; SETD7 (Set7/9), forward 5′-TCATTGATGTGCCTGAGCCCTA-3′ and reverse 5′-TCAGGGTGCGGATGCATTTGAT-3′; β-actin, forward 5′-GCACCACACCTTCTACAATGAGC-3′ and reverse 5′-TAGCACAGCCTGGATAGCAACG-3′.

### Assessment of Mitochondrial Membrane Potential

A day after seeding, cells were treated with 200 nM TMRE (Thermo Fisher Scientific, USA) for 30 min at 37°С in a CO_2_ incubator. Then, cells were washed in phosphate-buffered saline (PBS), detached with trypsin, and analyzed by flow cytometry (CytoFlex, Beckman Coulter, USA). Values of the median were used for calculations. Results were represented as the mean ± SEM of three experiments.

### Reactive Oxygen Species Detection Assay

The total reactive oxygen species (ROS) production was analyzed using the 2′,7′-dichlorodihydrofluorescein diacetate (H2DCFDA) substrate (Thermo Fisher Scientific, USA) in a final concentration of 50 µM. For detection, superoxide anions $\left({\text{O}}_{2}^{-}\right)$ dihydroethidium (DHE) (Thermo Fisher Scientific, USA) in a final concentration of 5 µM was used. The next day after seeding, cells were treated with H2DCFDA or dihydroethidium (DHE) for 30 min at 37°С in a CO_2_ incubator. Following the incubation, cells were washed in PBS, detached with trypsin, and analyzed by flow cytometry (FC) (CytoFlex, Beckman Coulter, USA). Values of the median were used for calculation. Results were represented as the mean ± SD of three experiments.

### Proliferation Assay

The proliferation analysis was performed using the xCELLigence technology (ACEA Biosciences, USA) according to the manufacturer’s instructions. A total of 20,000 cells were planted into each well of an ACEA E-plate 16 in triplicate. The cell index was registered every 10 min for 10–40 h. Results were represented as the mean ± SEM of three experiments.

### Cell Cycle Analysis

Flow cytometry analysis of the cell cycle was carried out. A total of 50,000 H1299 and H1975 cells, with Set7/9 KD or scramble, and A549 control and Set7/9 KO, were planted in triplicates. Two days after seeding, cells were harvested, washed once with PBS, and fixed in 70% ethanol at −20°C for 1 h. Then, 30 min staining of DNA content was carried out by using 50 μg/ml of PI (Invitrogen, USA) and 1 μg/ml RNase A (Thermo Fisher Scientific, USA). Samples were analyzed by a CytoFLEX (Beckman Coulter, USA) flow cytometer. Results were processed by CytoExpert software (Beckman Coulter, USA).

### Analysis of Glycolysis and Respiration

The SeaHorse energy profiling of lung cancer cell lines with either Set7/9 KD or scramble was carried out as described in (24) with small modifications. Briefly, 17,000 cells were seeded in SeaHorse 24-well plates a day prior to analysis. The SeaHorse Energy Phenotype test was used in accordance with the manufacturer’s recommendations. Stressor mix consisted of carbonyl cyanide p-(trifluoromethoxy)-phenyl-hydrazone (FCCP) and oligomycin (Agilent Technologies, USA) to achieve the final concentrations of 2 and 4 µM, respectively. Results are represented as the mean ± SEM. For further analysis of the impact of Set7/9 on glycolysis in detail, GlycoStress kit (Agilent Technologies, USA) was used according to the manufacturer’s instructions. The following concentrations were used in the glycolysis stress experiments: 10 mM of glucose, 4 µM of oligomycin, and 50 mM of 2-DG. Results are represented as the mean ± SEM.

### Microarray

The microarray gene expression study was carried out using Human Gene Expression 4x44K Microarrays and a Low Input Quick Amp Labeling Kit (Agilent Technologies, USA) according to the manufacturer’s instructions. RNA quality was assessed using a 2100 Bioanalyzer (Agilent Technologies, USA). One hundred nanograms of each RNA sample were used for cDNA synthesis and were simultaneously labeled with Cy-3. After purification, cDNA samples were hybridized with oligonucleotide probes on microarray slides for 18 h. The next day, the slides were washed and scanned. Data were analyzed by GeneSpring GX11.5 software.

### Bioinformatic Analysis of Lung Cancer Patients’ Survival Rates

The single and synergistic effect of SETD7 (Set7/9), SLC2A1 (GLUT1), HIF1A, HK2, and LDHA expression levels on the survival rate of lung cancer patients was determined by using Syntarget software as described in (25).

### Statistical Analysis

Data are represented as mean ± standard deviation (SD) or standard error of the mean (SEM) of at least three replicates. Statistical significance was analyzed using Student’s t-test. p < 0.05 was considered significant. p < 0.05 was denoted as * and p < 0.01 as **.

## Results

### Set7/9 Knockdown Upregulates c-Myc, HIF1A, and Genes of Glycolytic Enzymes in U2OS Human Osteosarcoma Cells

Set7/9 lysine methyltransferase is a well-known regulator of transcription (8, 10). Previously, we have shown that Tet-inducible Set7/9 knockdown in U2OS human osteosarcoma cells augmented the sensitivity of cells to genotoxic stress by upregulating the transcription of MDM2 (26).

To expand our observations on the role of Set7/9 in transcriptional regulation and to obtain the knowledge on global gene expression affected by Set7/9, we have carried out a microarray gene expression analysis of the same U2OS cells with Tet-inducible Set7/9 knockdown. We have treated U2OS with tetracycline for 2 days, followed by mRNA extraction, cDNA synthesis, labeling, and hybridization with oligonucleotide probes on microarray slides of the Illumina Human Gene Expression 4x44K Microarray Kit. The list of genes affected by Set7/9 KD is given in the **Supplementary Table 1**. The gene ontology analysis of differentially regulated genes in Set7/9 KD *versus* Set7/9 wt (U2OS pSuperior) cells revealed several functional pathways, among which the metabolic one was significantly represented. We focused on this pathway, since cancer metabolism is considered to be one of the hallmarks of cancer (27).

**Figure 1:**
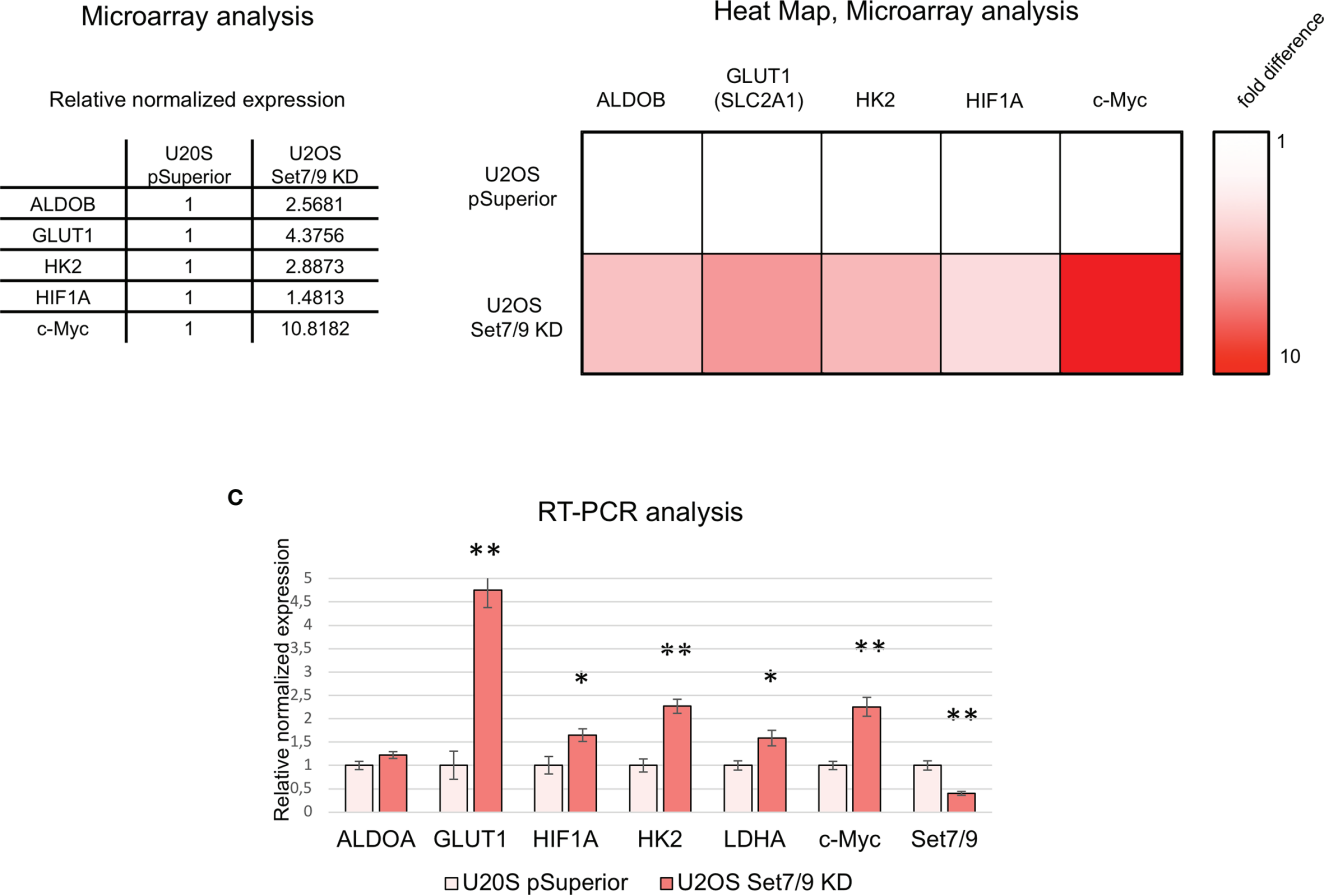
Microarray data analysis of U2OS Set7/9KD cells. **(A)** Relative expression of ALDOB, GLUT1, HK2, HIF1A, and c-Myc mRNAs in U2OS cells with tetracyclin-induced Set7/9 knockdown (tet-on system) compared to control U2OS pSuperior cells. Both cell lines were treated with doxycycline for 48 h prior to microarray analysis. **(B)** Heat map demonstrating ALDOB, GLUT1, HK2, HIF1A, and c-Myc mRNAs expression changes in Set7/9 KD U2OS cells compared to control U2OS pSuperior cells. **(C)** Validation of microarray data using quantitative RT-PCR: analysis of ALDOA, GLUT1, LDHA, HK2, HIF1A, c-Myc, and Set7/9 mRNAs expression in U2OS cells with tetracyclin-induced Set7/9 knock-down (Tet-on system) compared to control U2OS pSuperior cells. *p < 0.05; **p < 0.01.

Intriguingly, a number of genes encoding for the regulators of energy metabolism, e.g., glucose transporter [GLUT1 (SLC2A1)], glycolytic enzymes—hexokinase 2 (HK2) and aldolase (ALDOB)—and their key transcription regulators c-Myc and HIF1 were found among genes being significantly upregulated in Set7/9KD cells compared to control cells (U2OS pSuperior cells) (**Figures 1A, B**).

We have verified the microarray data by independent analysis using gene-specific real-time PCR. Indeed, Set7/9 knockdown augmented the mRNA levels of both c-Myc and HIF1A and their transcriptional targets—GLUT1, HK2, ALDOA, and LDHA (**Figure 1C**).

### Set7/9 Knockdown Upregulates c-Myc, HIF1A, and Glycolytic Genes in NSCLC Cells at Both Transcriptional and Protein Levels

**Figure 2:**
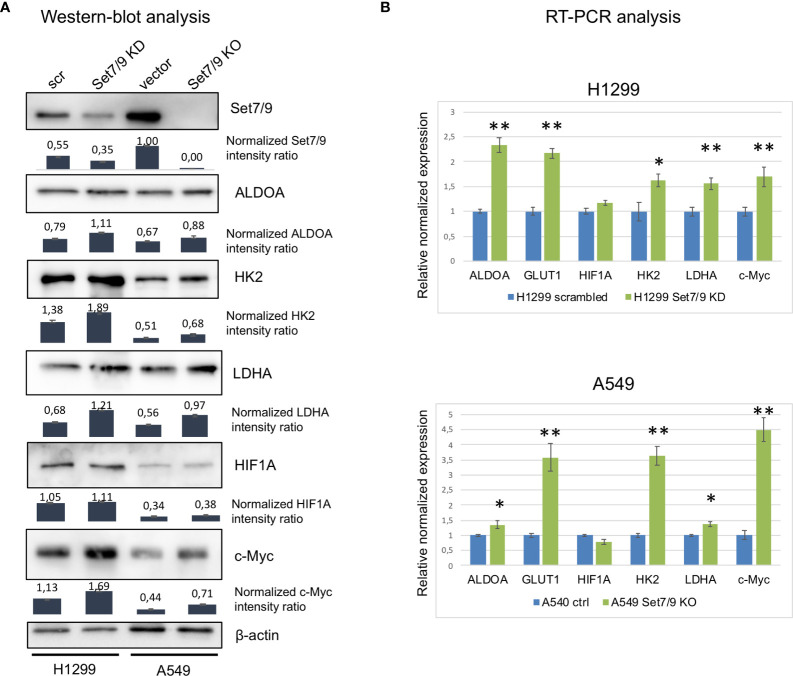
The effect of Set7/9 decrease on ALDOA, GLUT1, LDHA, HK2, HIF1A, and c-Myc levels. **(A)** Western blot analysis of ALDOA, LDHA, HK2, HIF1A, and c-Myc levels in NSCLC cell lines H1299 knockdown (Set7/9 KD) and A549 with Set7/9 knockout (Set7/9 KO) compared to control (scrambled or vector) cells. Densitometry analysis was performed on the basis of three measurements. Error bars indicate ± SD; **(B)** Quantitative RT-PCR: analysis of ALDOA, GLUT1, LDHA, HK2, HIF1A, and c-Myc mRNAs in the above cells. *p < 0.05; **p < 0.01.

We next asked if Set7/9 also affected the expression of c-Myc, HIF1A, GLUT1, and glycolytic enzymes in non-small cell lung cancer cell lines (NSCLCs). To this end, we stably suppressed Set7/9 expression [knock-down (KD) or knock-out (KO)] in three NSCLC cell lines with different p53 status [H1299 (p53-null), A549 (wild-type p53), and H1975 (mutant R273H p53)] (**Figure 2A** and **Supplementary Figure S1**). The mRNA level of c-Myc, HIF1A, GLUT1, HK2, ALDOA, and LDHA were assessed by real-time PCR (**Figure 2B** and **Supplementary Figure S1**). The results shown in **Figure 2A** demonstrate that, in general, the suppression of Set7/9 in NSCLC cells augmented the expression of energy metabolism factors similar to that observed in USOS osteosarcoma cells. Western blots were also consistent with RT-PCR results, i.e., attenuated levels of Set7/9 resulted in the increased production of the respective proteins, albeit to a different extent. Notably, the effect of Set7/9 was more pronounced in p53-negative cells (H1299) compared to A549 (p53-positive cells) or H1975 cell (p53 mut) (**Figure 2A** and **Supplementary Figure S1**). Furthermore, we found that the protein level of HIF1A was not significantly affected by Set7/9 in H1299 and A549 cells but was altered in H1975 cells with mutant p53 (**Figure 2** and **Supplementary Figure S1**, respectively).

### Set7/9 Knockdown Upregulates Mitochondrial Membrane Potential, Glycolysis, and Respiration

c-Myc and HIF1A are the two well-known master regulators of metabolic networks including energy metabolism, deregulations of which are recognized now as one of the “hallmarks of cancer.” Enhanced glycolysis in malignant cells is associated with tumor progression, migration, invasion, and resistance against chemo- and radiotherapy. Since our results suggested that ablation of Set7/9 caused an increase in c-Myc, GLUT1, and several glycolytic enzymes both at the mRNA and protein levels, we decided to examine whether Set7/9 affected the MMP, glycolysis, and respiration.

**Figure 3:**
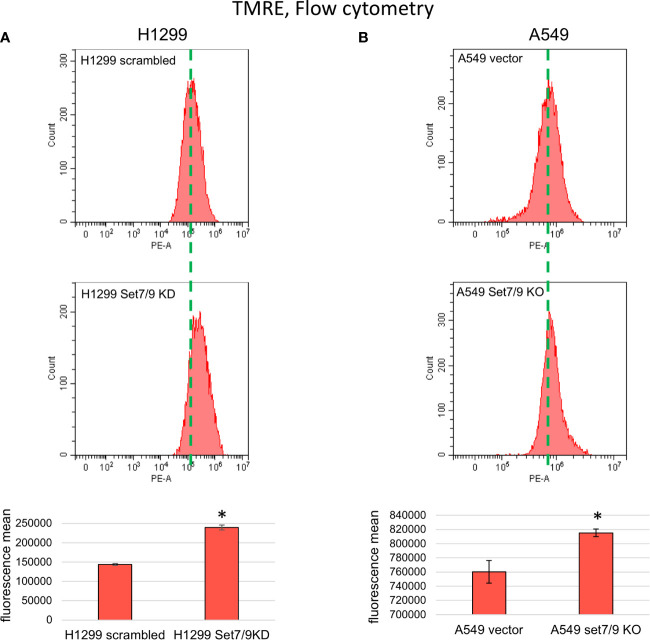
The effect of Set7/9 decrease on mitochondrial membrane potential (MMP) of NSCLC cell lines H1299, A549 and H1975. FC analysis of TMRE staining of NSCLC cell lines **(A)** H1299 and **(B)** A549 with Set7/9 knockdown (Set7/9 KD) and knockout (Set7/9 KO) compared to control (scrambled or vector) cells. *p < 0.01.

First, we stained H1299 and A549 cell lines with Set7/9 KD or Set7/9 KO, respectively, with the TMRE agent to measure the levels of MMP intensity. The respective parental control cell lines with wild-type Set7/9 were also used in the experiment. Cell lines with Set7/9 KD or KO increased the MMP level in the range of 10–60% depending on NSCLC cell line (**Figures 3A, B** and **Supplementary Figure S2A**). Importantly, the most pronounced effect was observed in p53-negative H1299 cells.

To confirm that the effect of Set7/9 ablation on MMP was not dependent on the p53 status, we used H1299 cell lines with Tet-inducible expression of wild-type p53 (p53wt) or mutant p53 R273H (p53mut) proteins, and control cells with knocked down Set7/9. We showed that in all three H1299-derived cell lines (control, p53wt, and p53mut), suppression of Set7/9 led to an increase in MMP levels (**Supplementary Figure S3**).

In general, malignant cells have 1.5–2 times higher MMP levels than their non-malignant counterparts, which is the consequence of an elevated energy metabolism. The level of MMP enhancement is associated with the degree of malignancy of neoplastic cells, including enhanced resistance to aggressive conditions and elevation of their metastatic potential (28).

**Figure 4:**
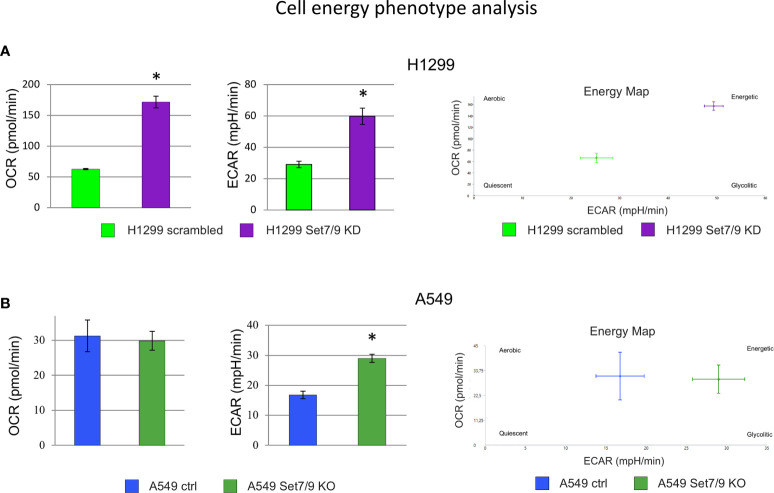
The effect of Set7/9 decrease on cell energy phenotype of NSCLC cell lines H1299, A549 and H1975 using SeaHorse system. The analysis of the oxygen consumption rate (OCR) and extracellular acidification rate (ECAR) of NSCLC cell lines **(A)** H1299 and **(B)** A549 with Set7/9 knockdown (Set7/9 KD) and knockout (Set7/9 KO) compared to control (scrambled or vector) cells. *p < 0.01.

Next, we used the SeaHorse energy profiling technology to study the impact of Set7/9 KD on glycolysis and respiration. Using the SeaHorse Energy Phenotype kit, we showed that Set7/9 KO upregulated only ECAR (glycolysis) in p53-positive A549 cells, whereas p53-negative H1299 cells and p53-mutant H1975 cells with Set7/9 knockdown displayed elevated levels of both glycolysis and respiration compared to control cells (**Figures 4A, B** and **Supplementary Figure S2C**).

Using the SeaHorse GlycoStress kit, we have further analyzed the impact of Set7/9 on several parameters of glycolysis (**Supplementary Figures S4–S6**). The attenuation of Set7/9 expression upregulated both glycolysis and respiration in H1299 and H1975 cell lines (**Supplementary Figures S4, S5**). On the contrary, only glycolysis was augmented in A549 cells upon Set7/9 attenuation, while respiration did not change significantly (**Supplementary Figure S6**). These results correlate with the data obtained using the SeaHorse Energy Phenotype kit (**Figure 4B**).

A previous study by Shen et al. showed that Set7/9 acts as a negative regulator of β-catenin in response to ROS (15). Importantly, β-catenin affects c-Myc expression, which in turn regulates the expression of glycolytic genes. Thus, it was plausible that Set7/9 mediated its effects on these genes through the β-catenin/c-Myc axis. To test this hypothesis, we assessed the effect of Set7/9 suppression on β-catenin levels in the NSCLC cell lines and observed no correlation between β-catenin levels and the status of Set7/9 (**Supplementary Figure S7A**).

Furthermore, we have tested the intracellular concentration of ROS in these cell lines. Using DHE staining, we have shown that the level of superoxide anions (O2−) was higher in all three Set7/9-deficient cell lines compared to control lines (**Supplementary Figure S7B**). However, the total amount of ROS species was either the same or lower in Set7/9 KD or Set7/9KO cells (**Supplementary Figure S7C**) compared to control cells. Since the intracellular superoxide anions content correlates with the activity of mitochondria (29, 30), this result further confirms the effect of Set7/9 suppression on mitochondrial activity.

### Set7/9 KD Enhances the Proliferation of NSCLC Cell Lines

**Figure 5:**
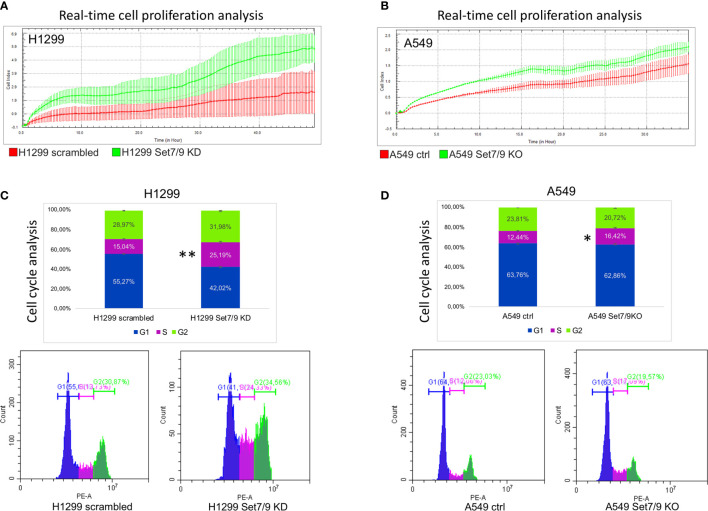
The effect of Set7/9 downregulation on the proliferation and cell cycle of NSCLC cell lines H1299 and A549. **(A, B)** The analysis of cell proliferation in real time, using the xCelligence system, of NSCLC cell lines **(A)** H1299 and **(B)** A549 with Set7/9 knockdown (Set7/9 KD) and knockout (Set7/9 KO), compared to control (scrambled or vector) cells. **(C, D)** The analysis of the cell cycle phases’ distribution of NSCLC cell lines **(C)** H1299 and **(D)** A549 with Set7/9 knockdown (Set7/9 KD) and knockout (Set7/9 KO) compared to control (scrambled or vector) cells. *p < 0.05; **p < 0.01.

The increased glycolysis is usually associated with the enhanced proliferation (31) and aggressiveness of tumors. Thus, we decided to study the effect of Set7/9 attenuation on the proliferation of the NSCLC cell lines. To address this question, we used the xCelligence platform. As shown in **Figures 5A, B** and **Supplementary Figure S8A**, all three NSCLC cell lines with Set7/9 KD/KO displayed augmented proliferation rates from 1.2- to 2-fold, compared to their respective controls. These results are in agreement with our data on the stimulatory effect of Set7/9 KD on glycolysis and respiration.

These results prompted us to assess the effect of Set7/9 on the cell cycle. To this end, we analyzed the cell cycle distribution of H1299, A549, and H1975 with different Set7/9 status. We observed a significant increase in S-phase in Set7/9 KD/KO cells in all three cell lines investigated in comparison to control cells (**Figures 5C, D** and **Supplementary Figure S8B**). These results, taken together with the results on proliferation obtained by xCelligence, strongly suggest that either the attenuation or ablation of Set7/9 upregulates proliferation rates of H1299, A549, and H1975 cells.

### Expression Levels of Set7/9, HIF1A, GLUT1, HK2, and LDHA Correlate With Survival Rates of Lung Cancer Patients

Increased rates of glycolysis in tumor cells are inversely associated with patient survival (32). We sought to investigate whether there are correlations between the expression levels of glycolytic genes HIF1A, GLUT1, HK2, and LDHA, in lung cancer patients and the rates of the patients’ survival. We carried out a bioinformatic analysis of several cancer samples databases using an algorithm previously described in SynTarget software (25).

**Figure 6:**
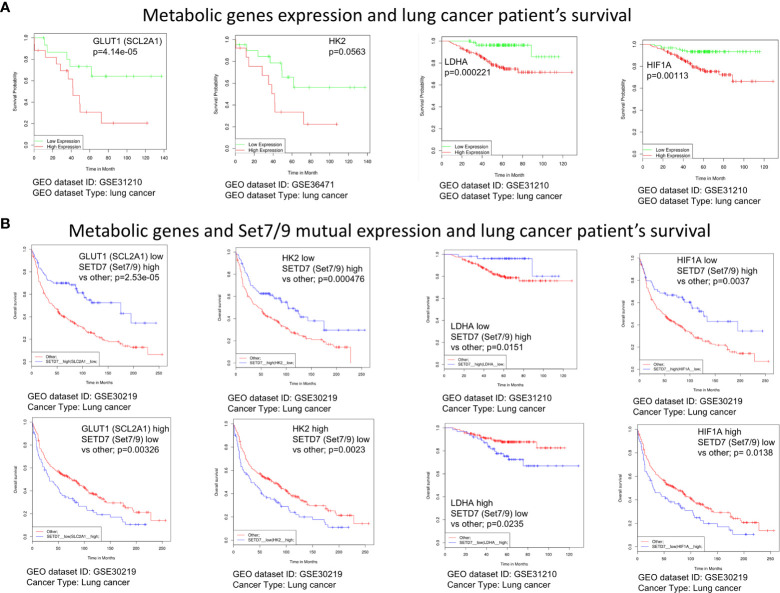
The expression level of Set7/9, HIF1A, GLUT1, HK2, and LDHA affects survival rate of lung cancer patients. **(A)** The bioinformatic analysis of the association of GLUT1 (SCL2A1), LDHA, HK2, and HIF1A expression levels with NSCLC patients’ survival. **(B)** The bioinformatic analysis of the GLUT1 (SCL2A1), LDHA, HK2, and HIF1A and Set7/9 (SETD7) mutual expression effect on NSCLC patients’ survival.

Kaplan–Meier plots (**Figure 6A**) demonstrate that the high levels of HK2, SLC2A1 (GLUT1), LDHA, and HIF1a expression were associated with poor survival of lung cancer patients. Moreover, low expression of Set7/9 together with high expression of each of aforementioned genes is also associated with poor outcome. On the contrary, high levels of Set7/9 expression in conjunction with low expression of HK2, SLC2A1 (GLUT1), LDHA, and HIF1A are associated with increased survival of patients (**Figure 6B**).

These observations reinforce our hypothesis that Set7/9 may have an impact on survival of cancer patients *via* regulating the energy metabolism of NSCLC.

## Discussion

A number of Set7/9 non-histone substrates, including p53 (33), MDM2 (34), E2F1 (16), STAT3 (21), affect proliferation, apoptosis, and resistance of NSCLC cells against chemotherapeutics.

In the present manuscript, we have provided evidence that the knockdown of Set7/9 methyltransferase in human NSCLC cell models upregulates a number of glycolytic enzymes (hexokinase, aldolase, and lactate dehydrogenase) and their key transcriptional activators—oncogenes c-Myc and HIF1A—at both transcriptional and protein levels.

Enhanced glycolysis has been a well-known feature of malignant cells since Otto Warburg’s pioneering works were published in 1925 (35). The so-called “Warburg effect” is part of metabolic reprogramming, which is considered now as one of the “hallmarks of cancer” (36). There are at least three main reasons why malignant cells benefit from glycolysis (37–39). First of all, glycolysis fuels biosynthetic anabolic pathways of rapidly proliferating cells by diverting glucose flux towards pentose-phosphate pathways and one-carbon metabolism. The latter are highly required for the synthesis of DNA, lipids, S-adenosyl methionine (the main donor of methyl groups), glutathione (an important factor of red-ox homeostasis), etc. Second, glycolysis helps neoplastic cells to fine-tune their interaction with the microenvironment by manipulating cancer-associated fibroblasts (CAFs). Finally, glycolysis provides extracellular acidification that protects malignant cells from the attack of immune cells, which are not effective at low pH.

c-Myc and HIF1A are master regulators of metabolic networks. They upregulate and coordinate glycolysis, respiration, one-carbon metabolism, and the metabolism of glutamine and lipids upon tumorigenesis (28, 40, 41). It is important to note that HIF1A and c-Myc are both known to regulate the expression of tumor-specific isoforms of glycolytic proteins such as GLUT1, HK2, PKM, and LDHA, thereby diminishing the Warburg effect in cancer cells (42–44). Moreover, both c-Myc and HIF1A play known roles in the upregulation of metabolic networks and the proliferation of NSCLC (23, 39, 45, 46).

Previously, two research groups have shown that Set7/9-mediated methylation of HIF1A and HIF2A leads to their destabilization and negatively regulates their transcriptional activity (47). In contrast, our present results suggest that Set7/9 affects HIF1A expression only at the level of mRNA in H1299 and A549 cells and only modestly on the protein level in H1975. Given that these three cell lines have different p53 statuses, it is unlikely that the effect of Set7/9, or lack thereof, was a p53-dependent effect. It is possible that the upregulation of HIF1A observed on the level of mRNA in the absence of Set7/9 could also be detected on the protein level should those cells be treated with hypoxia. Future experiments under hypoxic conditions should clarify this possibility. Nevertheless, in the present study, we have directly shown that the ablation of Set7/9 in several NSCLC cell lines upregulated glycolysis and respiration even in the absence of hypoxia (i.e., in normoxic conditions) (**Figure 4** and **Supplementary Figure S2**). These effects were likely due to the increased expression of the key glycolytic genes.

Importantly, HIF1A and c-Myc share common target genes such as HK2, ENO1, and LDHA, whose products are involved in glycolysis. In fact, most genes coding for glycolytic enzymes have in their promoter regions consensus binding sequences for both HIF1A and c-Myc (48). Thus, it is plausible that Set7/9 controls glycolysis indirectly, by affecting c-Myc expression. In support of this assumption is the fact that A549 and H1299 cells with suppressed Set7/9 displayed an increased expression of c-Myc both at the transcriptional and protein levels.

Among various malignancies, elevated glycolysis is associated with increased proliferation (49), metastasis (50–52), increased resistance to chemotherapy (53–55), and a poor survival prognosis (32).

In the present study, we demonstrated that the ablation of Set7/9 augmented the levels of HIF1A, c-Myc, and several genes coding for glycolytic enzymes. This points to Set7/9 as a potential tumor suppressor. Along with this notion, studies from several groups including ours also suggest that Set7/9 plays antiproliferative and tumor-protective roles in various cancers (7, 15, 56, 57), including NSCLC (21, 58).

In a number of works, Set7/9-mediated tumor suppressive effects have been achieved through p53 stabilization and activation. Indeed, we and others showed that Set7/9 is able to directly methylate the p53 protein, which leads to p53 stabilization and nuclear translocation and enhances the expression of p53 target genes (11, 59). Additionally, it was demonstrated that Set7/9 contributes to p53 stability *via* inactivation of SIRT1 by its methylation. Thus, the Set7/9-mediated methylation of SIRT1 prevented p53 deacetylation and hence increased p53 transactivation under genotoxic stress (60). Here, we demonstrate that Set7/9 acts as a regulator of glycolysis in NSCLC cells regardless of the p53 status. These results contribute to a better understanding of the p53-independent role of Set7/9 in tumorigenesis.

However, several papers on the role of Set7/9 in intestinal regeneration argue against the tumor-suppressive role of Set7/9. Apparently, being part of a YAP/AXIN1/β-catenin complex Set7/9 facilitates Wnt-induced nuclear accumulation of β-catenin, a known oncogenic protein (61).

Thus, the role of Set7/9 in tumorigenesis is likely to be cell tissue dependent. Future studies should discern the molecular mechanisms involving Set7/9 that affect tumor growth and metastases. Our results presented in this study indicate that Set7/9 is a potentially important regulator of metabolic networking in NSCLC.

## Data Availability Statement

All datasets presented in this study are included in the article/**Supplementary Material**. The proceeded microarray data was published in the Mendeley Datasets Repository (doi: 10.17632/hkgfsz9yhn.1 https://data.mendeley.com/datasets/hkgfsz9yhn/1).

## Author Contributions

AD, OS, and NB designed experiments and wrote the manuscript. AD and OS carried out majority of the experiments. OF and AZ participated in MMP assays. AP and EB participated in the establishment of cell lines and performed ROS analysis. AK participated in experiments using SeaHorse. LL performed microarray analysis. All authors contributed to the article and approved the submitted version.

## Funding

The authors acknowledge the support from RSF grant no. 19-75-10059. The shared research facility “Vertebrate cell culture collection” is supported by the Ministry of Science and Higher Education of the Russian Federation #075-15-2021-683.

## Conflict of Interest

The authors declare that the research was conducted in the absence of any commercial or financial relationships that could be construed as a potential conflict of interest.

## Publisher’s Note

All claims expressed in this article are solely those of the authors and do not necessarily represent those of their affiliated organizations, or those of the publisher, the editors and the reviewers. Any product that may be evaluated in this article, or claim that may be made by its manufacturer, is not guaranteed or endorsed by the publisher.

## Abbreviations

MMP, mitochondrial membrane potential; NSCLC, non-small cell lung cancer; TMRE, tetramethylrhodamine, ethyl ester; ECAR, extracellular acidification rate; OCR, oxygen consumption rate.
