# Supplementary material for: p53-Independent Effects of Set7/9 Lysine Methyltransferase on Metabolism of Non-Small Cell Lung Cancer Cells
Source: Front Oncol. 2021 Oct 6;11:706668. doi: 10.3389/fonc.2021.706668 (PMC8528242; doi:10.3389/fonc.2021.706668)
Supplement: Supplementary file 1 [file Presentation_1.pptx]

## Slide 1
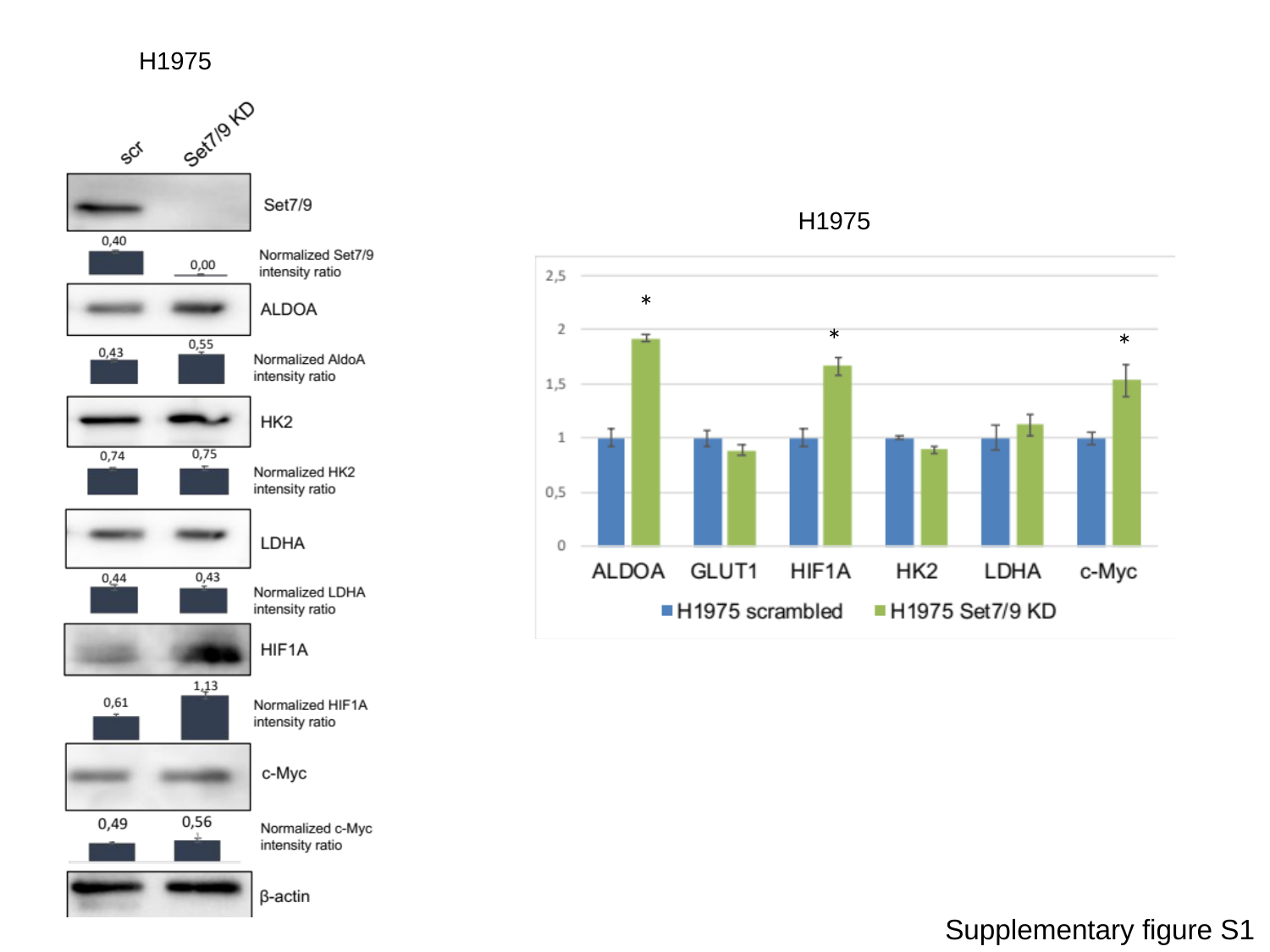

H1975
H1975
*
*
*
Supplementary figure S1

## Slide 2
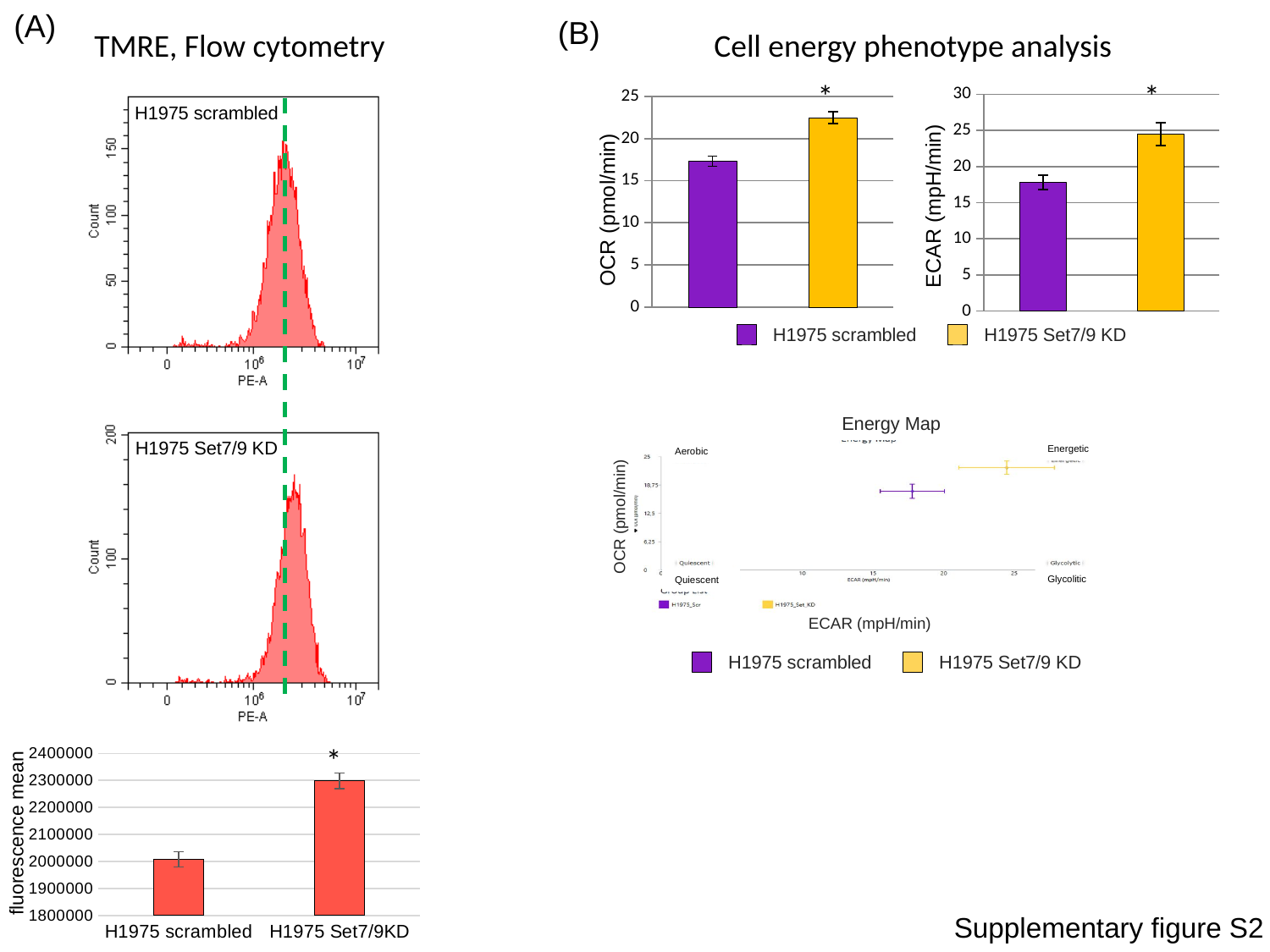

(A)
(B)
TMRE, Flow cytometry
Cell energy phenotype analysis
*
*
### Chart
| Category | |
|---|---|
| H1975_Scr | 17.8 |
| H1975_KD | 24.47 |
### Chart
| Category | |
|---|---|
| H1975_Scr | 17.3 |
| H1975_KD | 22.5 |
H1975 scrambled
OCR (pmol/min)
ECAR (mpH/min)
H1975 scrambled
H1975 Set7/9 KD
Energy Map
H1975 Set7/9 KD
Energetic
Aerobic
OCR (pmol/min)
Glycolitic
Quiescent
ECAR (mpH/min)
H1975 scrambled
H1975 Set7/9 KD
*
### Chart
| Category | |
|---|---|
| H1975 scrambled | 2007422.95 |
| H1975 Set7/9KD | 2298704.525 |fluorescence mean
Supplementary figure S2

## Slide 3
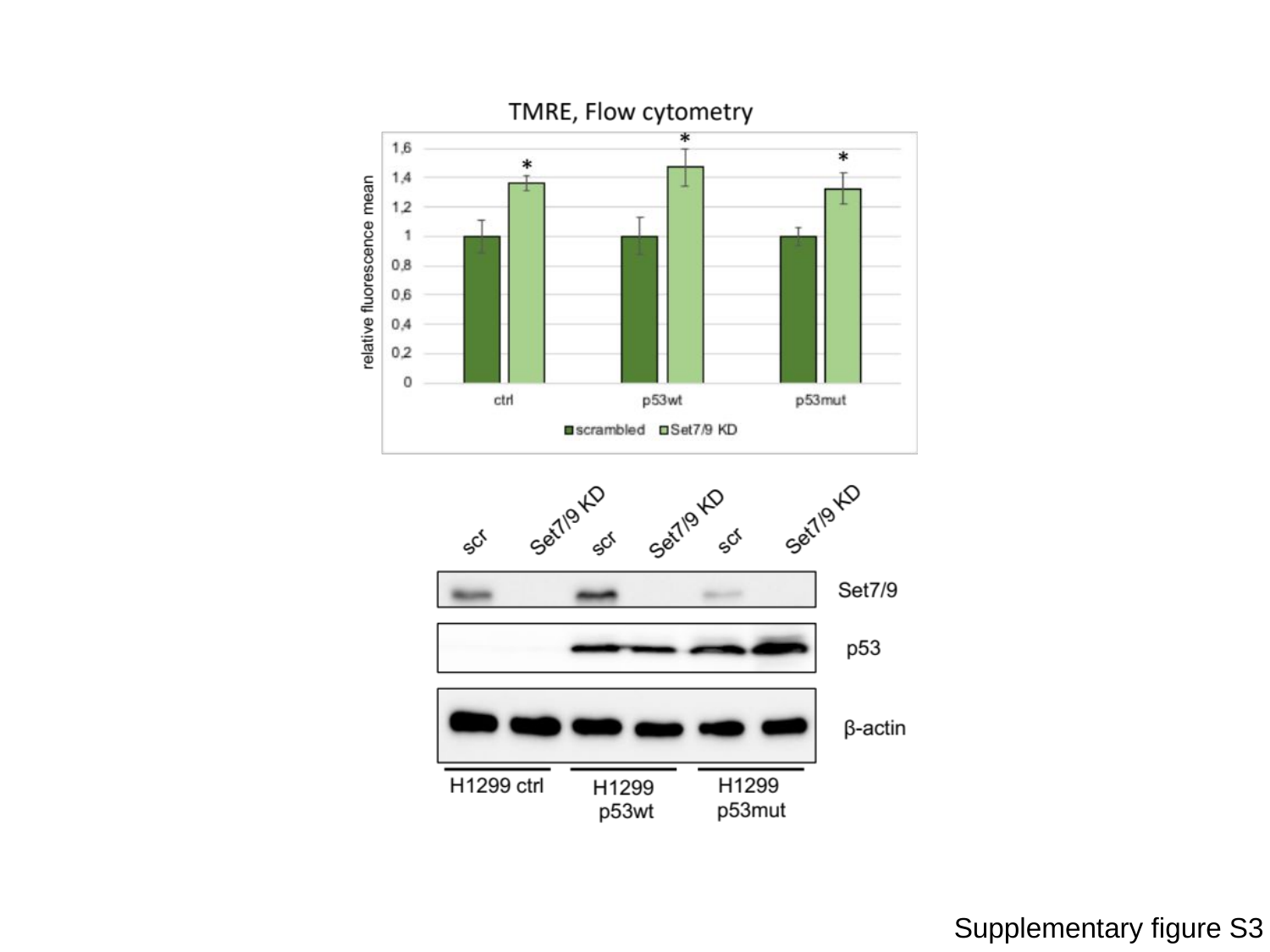

Supplementary figure S3

## Slide 4
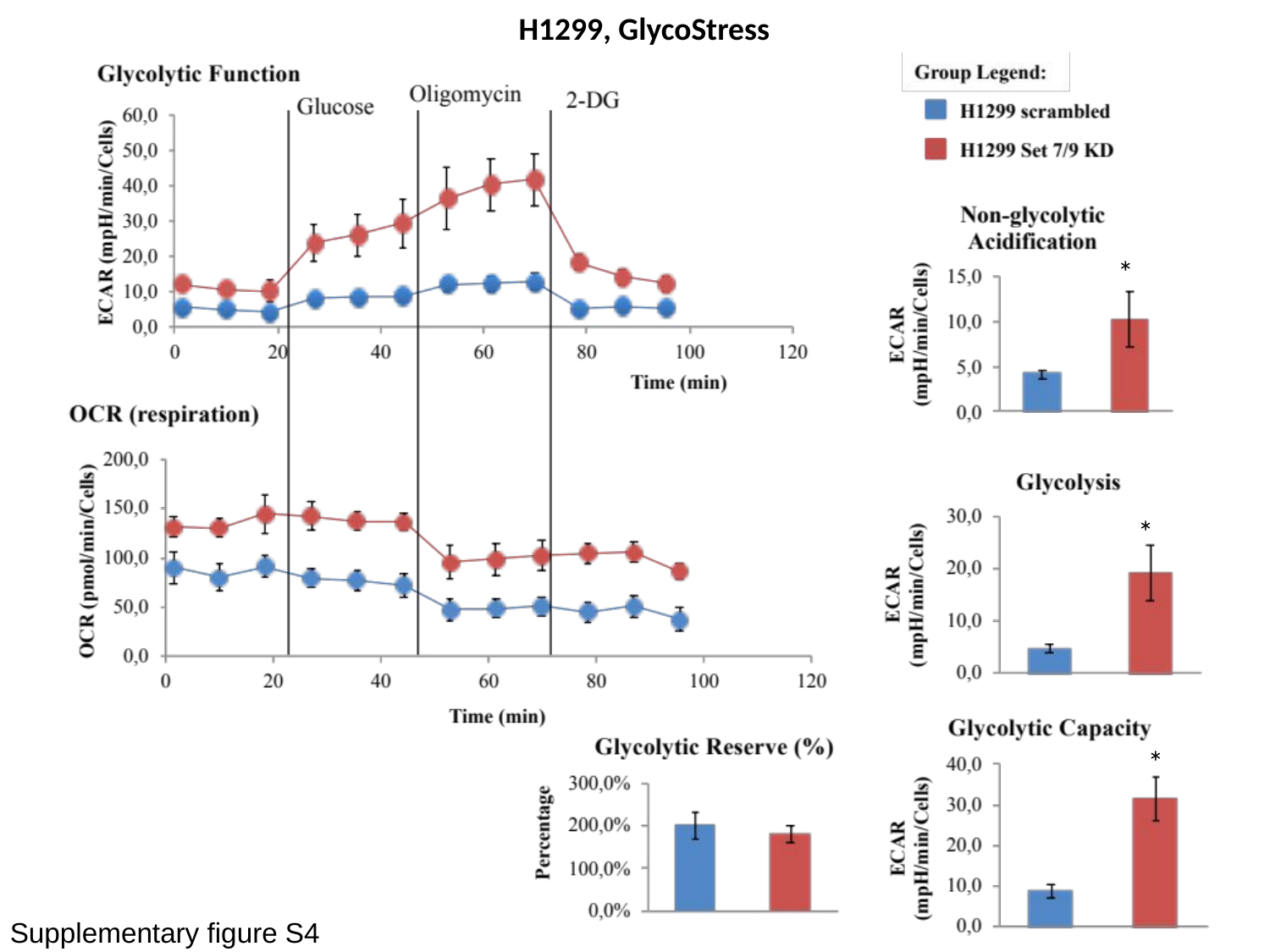

H1299, GlycoStress
*
*
*
Supplementary figure S4

## Slide 5
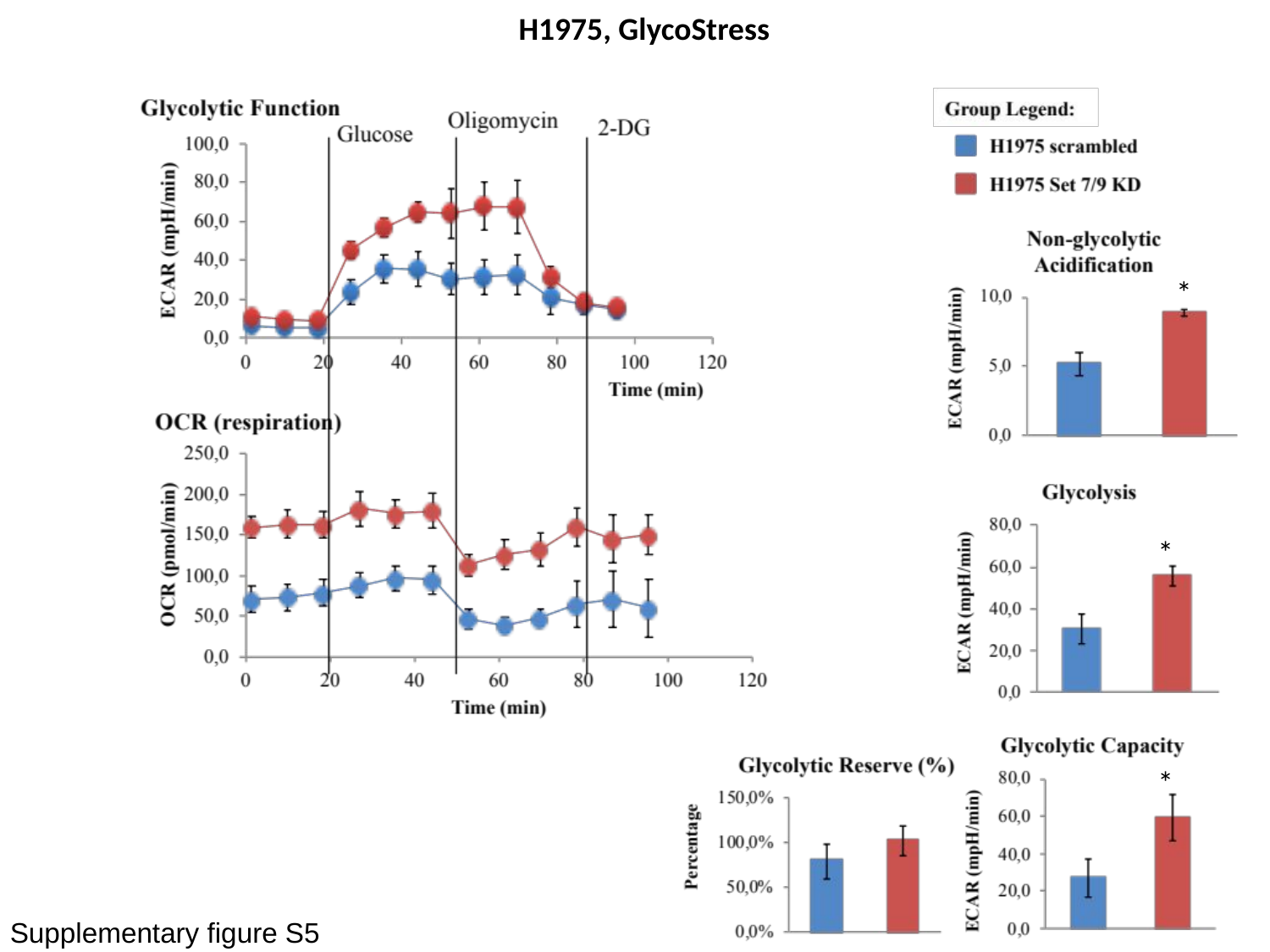

H1975, GlycoStress
*
*
*
Supplementary figure S5

## Slide 6
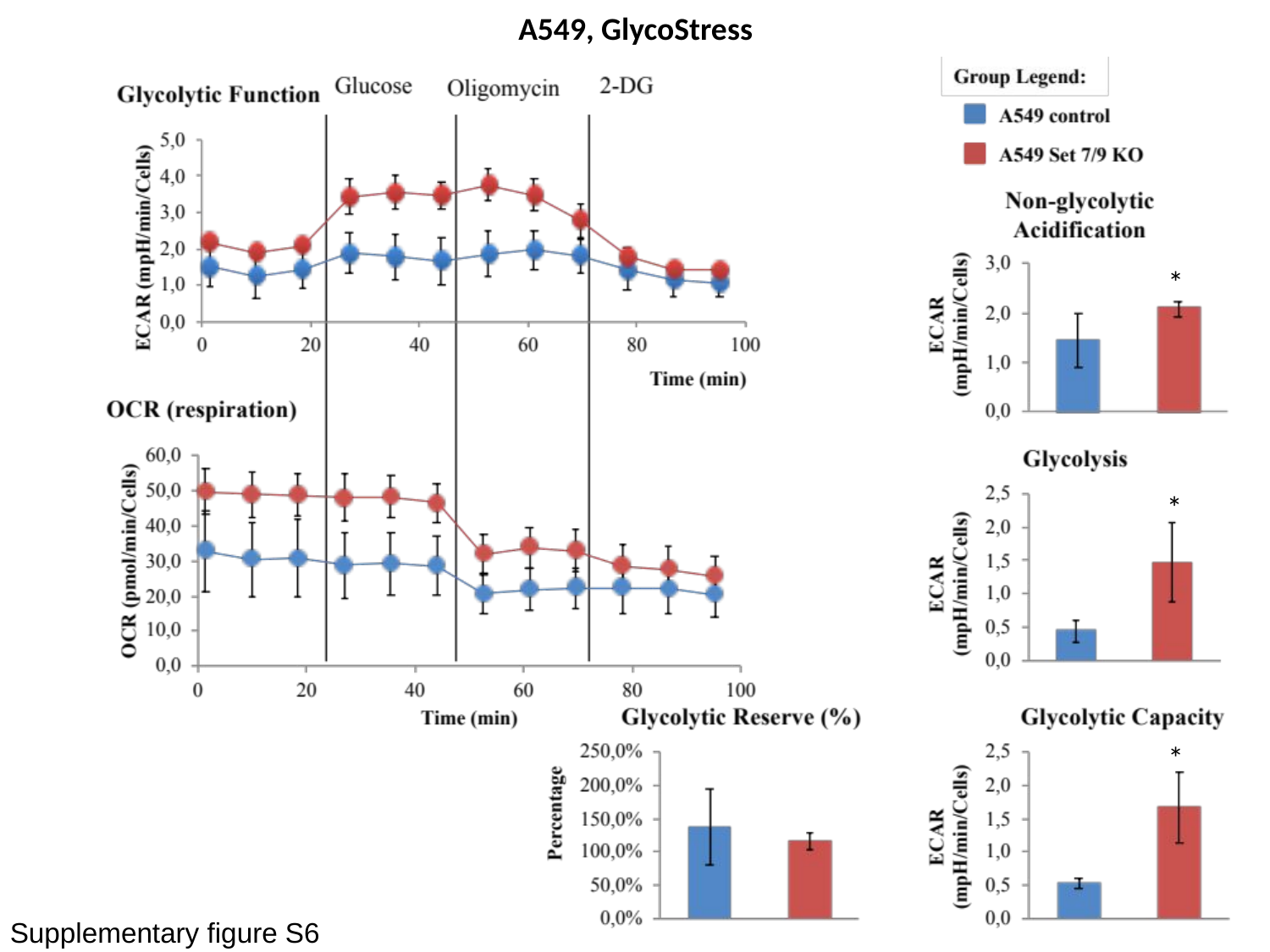

A549, GlycoStress
*
*
*
Supplementary figure S6

## Slide 7
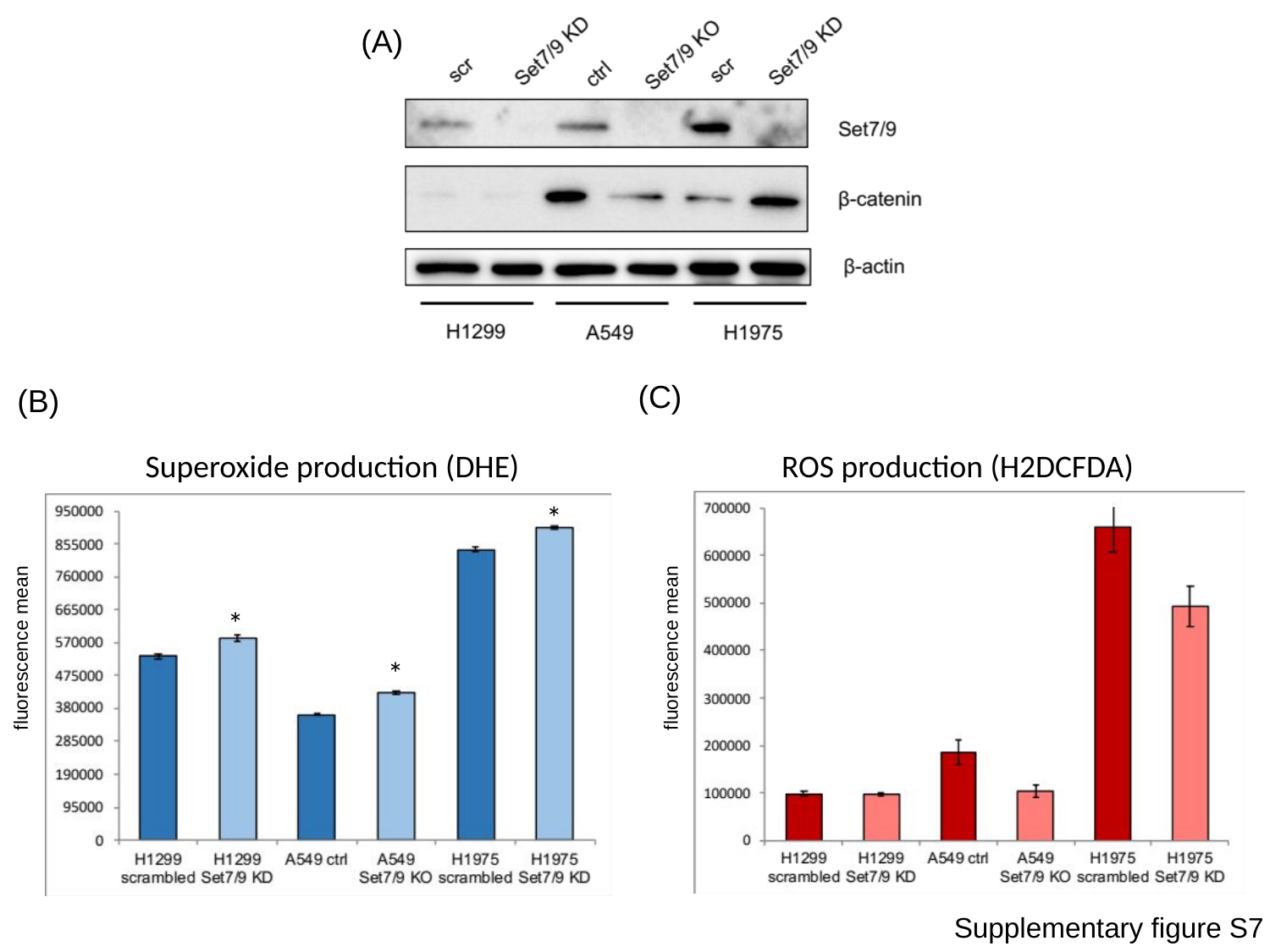

(A)
(C)
(B)
Superoxide production (DHE)
ROS production (H2DCFDA)
*
*
fluorescence mean
fluorescence mean
*
Supplementary figure S7

## Slide 8
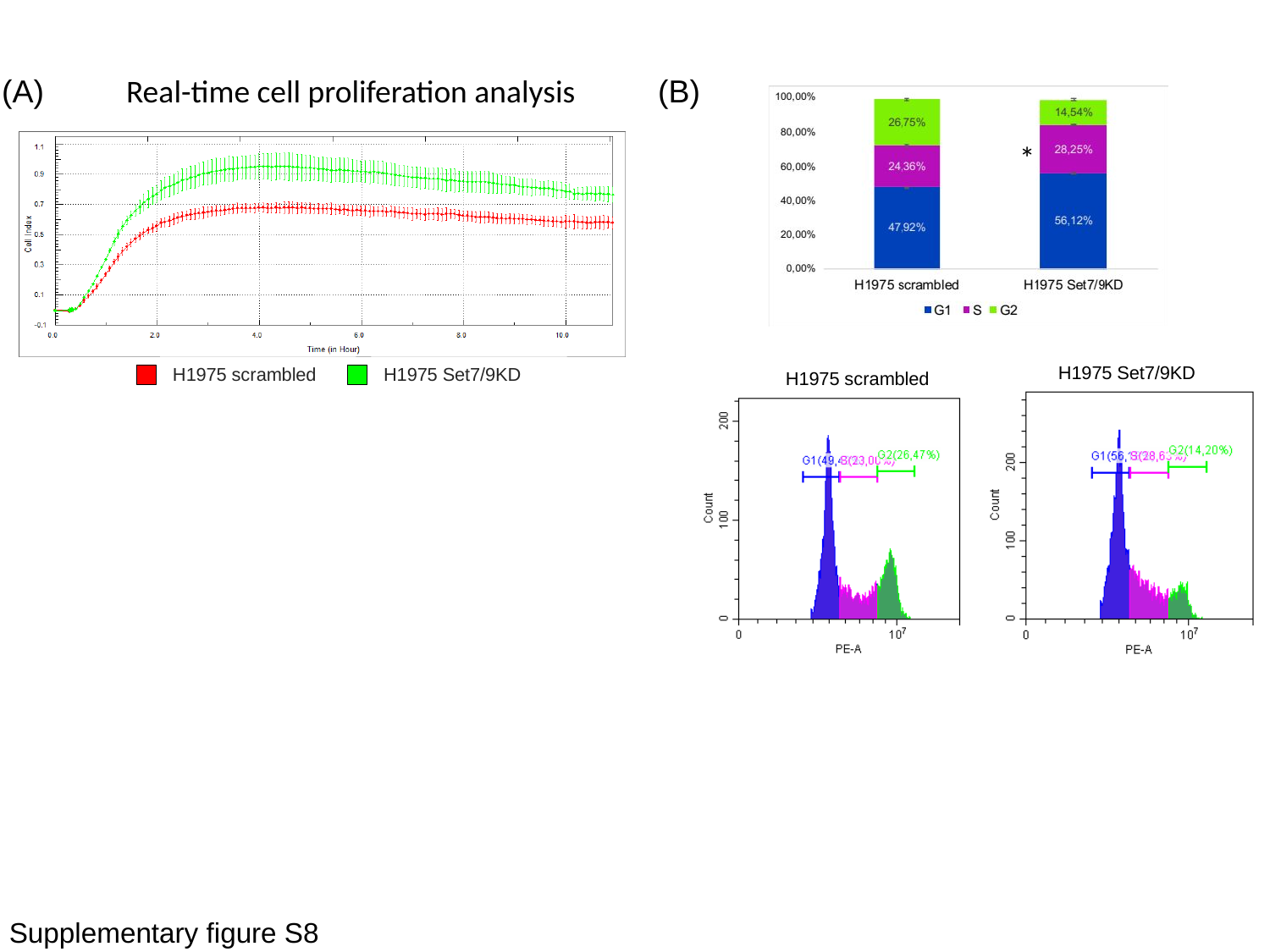

(A)
Real-time cell proliferation analysis
(B)
*
H1975 Set7/9KD
H1975 scrambled
H1975 Set7/9KD
H1975 scrambled
Supplementary figure S8
